# Aggressive challenging behavior in adults with intellectual disability: An electronic register-based cohort study of clinical outcome and service use

**DOI:** 10.1192/j.eurpsy.2022.2336

**Published:** 2022-11-02

**Authors:** James Smith, R. Asaad Baksh, Angela Hassiotis, Rory Sheehan, Chengcheng Ke, Tsz Lam Bambi Wong, PETAL Investigators, André Strydom

**Affiliations:** 1 South London and Maudsley NHS Foundation Trust, London, United Kingdom; 2 Institute of Psychiatry, Psychology and Neuroscience, King’s College London, London, United Kingdom; 3 The LonDownS Consortium, London, United Kingdom; 4 Division of Psychiatry, UCL, London, United Kingdom; 5 Camden Learning Disability Service, London, United Kingdom

**Keywords:** Intellectual disability, adults, aggressive challenging behaviour, aggression, adverse outcome, cohort study

## Abstract

**Background:**

Aggressive challenging behavior in people with intellectual disability is a frequent reason for referral to secondary care services and is associated with direct harm, social exclusion, and criminal sanctions. Understanding the factors underlying aggressive challenging behavior and predictors of adverse clinical outcome is important in providing services and developing effective interventions.

**Methods:**

This was a retrospective total-population cohort study using electronic records linked with Hospital Episode Statistics data. Participants were adults with intellectual disability accessing secondary services at a large mental healthcare provider in London, United Kingdom, between 2014 and 2018. An adverse outcome was defined as at least one of the following: admission to a mental health hospital, Mental Health Act assessment, contact with a psychiatric crisis team or attendance at an emergency department.

**Results:**

There were 1,515 patient episodes related to 1,225 individuals, of which 1,019 episodes were reported as displaying aggressive challenging behavior. Increased episode length, being younger, psychotropic medication use, pervasive developmental disorder (PDD), more mentions of mood instability, agitation, and irritability, more contact with mental health professionals, and more mentions of social and/or home care package in-episode were all associated with increased odds of medium-high levels of aggression. Risk factors for an adverse clinical outcome in those who exhibited aggression included increased episode length, personality disorder, common mental disorder (CMD), more mentions of agitation in-episode, and contact with mental health professionals. PDD predicted better outcome.

**Conclusions:**

Routinely collected data confirm aggressive challenging behavior as a common concern in adults with intellectual disability who are referred for specialist support and highlight factors likely to signal an adverse outcome. Treatment targets may include optimizing management of CMDs and agitation.

## Introduction

Intellectual disability is a lifelong condition characterized by impairment in cognition, language, and social ability [1] which affects approximately 1% of the global population [2]. Broadly defined challenging behavior, including self-injury, aggression, and stereotyped behavior, is estimated to affect up to one-quarter of people with intellectual disability [3, 4]. Aggressive challenging behavior includes verbal aggression and threats, physical violence, property damage, sexually aggressive, and self-injurious behavior. Although prevalence estimates vary depending on the population studied, method of assessment, and definition, aggressive challenging behavior has a point prevalence of approximately 10% amongst adults with intellectual disability and tends to persist over time, with an estimated 25% remission rate at 2 years [5–7]. Aggressive challenging behavior is a common reason for referral to health services of adults with intellectual disability and can have serious consequences including exclusion from services, admission to hospital, and contact with the criminal justice system [8, 9]. A study of physically aggressive challenging behavior [10] used data from the Leicestershire Intellectual Disability Register in the United Kingdom to examine associations with clinical outcomes. The findings included permanent exclusion from day activities in 4% of participants, police involvement in 10%, and family and carer stress in almost half of the carers (42%).

Previous studies that have explored factors associated with aggressive challenging behavior in adults with intellectual disability have shown male gender, comorbid autism spectrum disorder, communication impairment, and severe intellectual disability to be independently associated with higher levels of aggression [3, 6, 11, 12]. However, the evidence-base is relatively small, and findings are inconsistent across studies. Individuals who display aggressive challenging behavior may have contact with emergency and crisis services, but such associations have not been explored in the United Kingdom context, for example, being referred to a crisis team, presenting to the emergency department, or being assessed under the Mental Health Act in the United Kingdom for involuntary admission to hospital. However, research undertaken outside the United Kingdom shows that people with intellectual disability are more likely to visit emergency services [13] and that type of residence and not having a crisis plan, are associated with such visits.

In this study, we aimed to update the evidence on factors associated with, and outcomes of, aggressive challenging behavior in adults with intellectual disability by (a) investigating risk factors for aggressive challenging behavior and (b) identifying correlates of contact with crisis services that may be associated with an adverse clinical outcome (such as emergency department attendance or hospital admission) using a secondary care electronic register with record linkage.

## Method

### Data source

Data were obtained from the South London and Maudsley (SLaM) National Health Service (NHS) Foundation Trust in the United Kingdom. SLaM is one of the largest providers of mental healthcare in Europe, with a footprint covering approximately 1.5 million residents in four diverse south London boroughs. The Clinical Record Interactive Search (CRIS) system was established in 2008 to allow researchers access to deidentified structured and open-text data held in the electronic health record (EHR) used by all SLaM clinical services. CRIS can also provide data extracts through linkage with other sources such as Hospital Episode Statistics (HES) [14, 15]. Detailed information about the range of data linkages within CRIS is available [16].

CRIS data from EHRs can be extracted using diagnostic codes based on the International Classification of Mental and Behavioral Disorders-10 (ICD-10) [1], or structured fields, for example, for patient contacts by clinicians. In addition, data may be extracted from unstructured free text fields entered by clinicians on the patients’ EHR susing natural language processing (NLP) applications developed with General Architecture for Text Engineering was developed by the University of Sheffield, Sheffield, United Kingdom. The NLP processes free text fields of clinical documentation using a machine learning approach, to extract information from clinical notes [15, 17]. The study was registered with the CRIS oversight board in accordance with CRIS’s overarching ethical approvals for research use of extracted clinical data.

### Participants

Eligible patients were 18 years old or older at cohort entry and had a documented diagnosis of intellectual disability according to ICD-10 criteria (codes F70–F79) [1]. Participants must have had an episode of outpatient care including direct contact with a specialist intellectual disability mental health team within SLaM between January 1, 2014, and December 31, 2018. Very brief episodes of care recorded as lasting fewer than 14 days were not included in the sample on the basis that such episodes were likely to include only single assessments or inappropriate referrals. Episodes may have continued after the cut-off date but no data were included beyond December 31, 2018.

Patients could have multiple episodes within the study time period and each episode was considered independently of any others in the analysis. Overlapping episodes of care, where a person had two open episodes simultaneously, were removed according to the following rules: (a) if episodes overlapped completely, the shorter episode was discarded as all information would be included in the longer episode; (b) if care episodes partially overlapped, both episodes were included in the analysis and categorized as overlapping episodes. For example, a patient could have one episode which began on January 1, and finished on January 31, and during this time, the dataset also consisted of another episode of the same patient which began on January 14, and finished on March 2.

### Measures

#### Clinical diagnoses

Clinical diagnoses were extracted from structured fields within patients’ EHRs: substance misuse (ICD-10 codes: F10–F19), nonaffective psychotic disorders (F2*), bipolar/mania (F30–F31) and depression (F32–F39), neurotic and stress-related (F4*), personality disorders (F6*), pervasive developmental disorders (PDDs, F8*), and attention deficit hyperactivity disorder (ADHD, F90). We combined nonaffective psychotic disorders and bipolar/mania diagnoses into a severe mental illness (SMI) category and depression and neurotic and stress-related diagnoses into a common mental disorder (CMD) category. Diagnoses of selected medical comorbidities were also extracted; epilepsy (G40*), metabolic diseases (E70–E90), and congenital and chromosomal disorders (Q00–Q99).

#### Other clinical features

Relevant NLP applications were used to extract the following data from free text within EHR fields: mentions of aggression, mood instability, agitation, and irritability. We included these symptoms for two reasons: first, because mood instability is often present as symptom outside the confines of a specific mood disorder [18] and second, because irritability and agitation have been considered to lie on the pathway leading to aggressive challenging behavior [19]. Furthermore, these symptoms are associated with adverse clinical outcomes outside of diagnostic boundaries [20]. Finally, we extracted any mention of receiving social care support (defined as “instances of receiving current, recommended or planned general care package…a generic term relating to any social care intervention”) or home care (defined as “instances of home care/help, that is, help by someone who comes to assist the patient with activities of daily living”).

These NLPs have been developed and previously validated [17]. The NLP for aggression was further validated in 481 records for people with intellectual disability with a precision of 90.9% in extracting mentions of current or historic aggression. We then divided the aggression variable into four groups based on quantiles and distribution, to gain a better understanding of the sample in relation to frequency of aggression within a care episode: no aggression (0 mentions in episode); low frequency of aggression (1–3 mentions); moderate frequency of aggression (4–8 mentions); and high frequency of aggression (≥9 mentions).

#### Medication

Mentions of any psychotropic medication use were extracted using NLP searches. Medication was categorized according to the British National Formulary into hypnotics/anxiolytics, antipsychotics, antidepressants, medication for ADHD (BNF section 4.4), and antiepileptics. Thyroid medication, analgesics, and laxatives were combined into one category (treatments for common physical health conditions).

#### Contact with mental health professionals

The number of contacts with mental health professionals within the care episode (including nurses, psychologists, and psychiatrists) were obtained using structured data.

#### Clinical outcome

Data acquired by data linkage to HES in each episode was used to explore clinical outcome during an episode of care. An adverse outcome was defined as at least one of the following: (a) admission to a mental health hospital, (b) Mental Health Act assessment, (c) contact with a psychiatric crisis team, or (d) attendance at an emergency department.

### Statistical analysis

We used Fisher’s exact test for categorical variables and the Kruskal–Wallis test for continuous variables to describe the sample by frequency of aggressive challenging behavior and relationship to sociodemographic and clinical details.

We fitted mixed effects logistic regression models to the episode-level data to estimate which clinical and service-related variables were associated with aggression in-episode, reporting odds ratios (OR) and 95% confidence intervals (95% CI). Mixed effects logistic regressions were fitted over standard logistic regressions to account for some patients having more than one episode in the dataset, and therefore nonindependent data. For this analysis, we combined the no and low aggression groups and the medium and high groups to create a binary variable (no-low aggression or moderate-high aggression). In Model 1, demographic variables (age, sex, ethnicity, and level of intellectual disability), episode length in years, and the presence of any overlapping episodes were entered as covariates. In Model 2, all psychiatric, medical comorbidities (epilepsy, genetic, metabolic, or chromosomal disorder), clinical symptoms, and medication variables were included in addition, to examine potential associations with these clinical variables and the presentation of medium-high levels of aggressive challenging behavior in an episode. In Model 3, total number of contacts with mental health professionals and total number of mentions of a social care and/or a home care package in patient’s clinical notes during their episode were added to explore the relationship between service-related variables and the presentation of medium-high levels of aggression while adjusting for other covariates. To examine outcomes in those who displayed aggression, we fitted further mixed effects logistic regression models following the same procedure to examine risk factors of an adverse clinical outcome in patients who had at least one mention of aggression within an episode of care. All analyses were conducted using R [21].

## Results

### Description of study population

A total of 1,225 patients (477 females and 748 males) contributed a total of 1,515 episodes of care which met the inclusion criteria. The median age at the start of an episode was 37 years (interquartile range [IQR]: 27–55).

Episodes with high frequency of aggression were longer in duration (2 years 3 months) than those with lower frequency or no mention of aggression (no aggression, approximately 4 months; low, 8 months; and medium-high, 1 year 3 months). Episodes of high aggression were reported in younger adults (median age 31 years), in males and those with mild intellectual disability (Table 1).

**Table 1.** Demographic information of episodes of care split by frequency of aggression.

		No aggression in episode	Low aggression in episode	Moderate aggression in episode	High aggression in episode	*p*-value
*N*		496	519	249	251	
Median episode length in years (IQR)		0.36 (0.17–0.81)	0.67 (0.31–1.19)	1.25 (0.74–1.86)	2.26 (1.34–3.32)	<0.001
Median mention of aggression in episode (IQR)		0 (0–0)	2 (1–2)	5 (4–6)	15 (11–23)	<0.001
Intellectual disability level	Mild	137 (27.6)	199 (38.3)	84 (33.7)	106 (42.2)	<0.001
	Moderate	96 (19.4)	123 (23.7)	71 (28.5)	65 (25.9)	
	Severe/profound	58 (11.7)	62 (11.9)	29 (11.6)	43 (17.1)	
	Not specified/unknown	205 (41.3)	135 (26.0)	65 (26.1)	37 (14.7)	
Median age (IQR)		42 (30–57)	40 (29–57)	32 (36–53)	31 (25–50)	<0.001
Sex	Female	235 (47.4)	191 (36.8)	83 (33.3)	81 (32.3)	<0.001
	Male	261 (52.6)	328 (63.2)	166 (66.7)	170 (67.7)	
Ethnicity	White	243 (49.0)	291 (56.1)	121 (48.6)	114 (45.4)	<0.001
	Black	153 (30.8)	146 (28.1)	85 (34.1)	86 (34.3)	
	Asian	33 (6.7)	29 (5.6)	11 (4.4)	21 (8.4)	
	Mixed	11 (2.2)	19 (3.7)	10 (4.0)	13 (5.2)	
	Other	13 (2.6)	5 (1.0)	11 (4.4)	16 (6.4)	
	Not stated	43 (8.7)	29 (5.6)	11 (4.4)	1 (0.4)	
PDD diagnosis		122 (24.6)	175 (33.7)	122 (49.0)	148 (59.0)	<0.001
ADHD diagnosis		12 (2.4)	32 (6.2)	21 (8.4)	26 (10.4)	<0.001
Ever diagnosed with SMI		40 (8.1)	59 (11.4)	38 (15.3)	65 (25.9)	<0.001
Ever diagnosed with CMD		81 (16.3)	96 (18.5)	33 (13.3)	57 (22.7)	0.036
Ever diagnosed with a personality disorder		9 (1.8)	17 (3.3)	7 (2.8)	21 (8.4)	<0.001
Physical health medication		42 (8.5)	116 (22.4)	71 (28.5)	108 (43.0)	<0.001
Psychotropic medication		136 (27.4)	315 (60.7)	199 (79.9)	234 (93.2)	<0.001
Median mention of mood instability in episode (IQR)		0 (0–0)	0 (0–0)	0 (0–1)	1 (0–3)	<0.001
Median mention of agitation in episode (IQR)		0 (0–0)	1 (0–2)	3 (1–6)	10 (5–19)	<0.001
Median mention of irritability in episode (IQR)		0 (0–0)	0 (0–0)	0 (0–1)	1 (0–4)	<0.001
Ever diagnosed with epilepsy		48 (9.7)	59 (11.4)	29 (11.6)	39 (15.5)	0.13
Genetic, metabolic, or chromosomal disorders		56 (11.3)	67 (12.9)	34 (13.7)	32 (12.7)	0.78
Ever diagnosed with substance misuse		16 (3.2)	9 (1.7)	5 (2.0)	5 (2.0)	0.46
Mental Health Act assessment in episode		1 (0.2)	4 (0.8)	7 (2.8)	45 (17.9)	<0.001
Mental Health hospital admission in episode		2 (0.4)	1 (0.2)	4 (1.6)	37 (14.7)	<0.001
Contact with crisis team in episode		0 (0.0)	3 (0.6)	4 (1.6)	15 (6.0)	<0.001
Attendance at A&E in episode		126 (25.4)	156 (30.1)	103 (41.4)	161 (64.1)	<0.001
Median mental health professional contacts in episode (IQR)		1 (0–3)	2 (1–5)	5 (3–10)	15 (9–29)	<0.001
Median mention of social care package and/or home care package in episode (IQR)		0 (0–0)	0 (0–0)	0 (0–1)	1 (0–3)	<0.001

*Note.* Percentages are in parentheses unless otherwise indicated.

*Abbreviations*: ADHD, attention deficit hyperactivity disorder; A&E, accident and emergency; CMD, common mental disorder (depression and/or neurotic and stress-related disorders); IQR, interquartile range; Other ethnicity groups, Arab and other minority ethnicity groups; PDD, pervasive developmental disorder diagnosis; Physical health medication, thyroid and antithyroid drugs, analgesics, and laxatives; Psychotropic medication, hypnotics/anxiolytics, antipsychotics, antidepressants, ADHD, and antiepileptic medication; SMI, severe mental illness (nonaffective psychotic disorders and/or bipolar/mania).

Mood instability, irritability, and agitation were strongly associated with increasing occurrence of aggressive challenging behavior.

Nearly 60% of patient episodes in the high aggression group had a recorded diagnosis of PDD compared to 25% of those in the no aggression group. Nearly 93% of episodes in the high aggression group had a mention of psychotropic medication, 64% recorded an accident and emergency attendance, and a median of 1 (0–3) mention of social care and/or home care package in their clinical notes. Nearly 18% of episodes in the high aggression group had a recorded Mental Health Act assessment compared with just 0.2 and 0.8% in the no and low aggression group, respectively. Additionally, 43% of the high-aggression group had a mention of receiving medications for physical health conditions, compared with 8.5% in the no-aggression group. There were no statistically significant differences between aggression groups in terms of diagnoses for epilepsy, genetic, metabolic, or chromosomal disorders (Table 1). Therefore, these variables were excluded from further analysis.

### Factors associated with aggression

In Model 1, displaying medium-high levels of aggressive challenging behavior in-episode was strongly associated with being younger, male, and having longer episode duration (in years). Model 2 found that ethnicity (particularly those from the “other” group), psychotropic medication use, a PDD diagnosis, episode length, mood instability, irritability, and agitation were significantly associated with aggressive challenging behavior. The addition of variables related to service use in Model 3 did not alter the results from Model 2, but added contact with mental health professionals and mentions of social care and/or home care package was significantly associated with an increased likelihood of displaying medium-high levels of aggressive challenging behavior. Details of the analyses are presented in Table 2.

**Table 2.** Results of the adjusted modeling examining variables associated with moderate-high levels of aggression in episode.

		Model 1		Model 2		Model 3	
		OR (95% CI)	*p*-value	OR (95% CI)	*p*-value	OR (95% CI)	*p*-value
Episode length in years		4.92 (3.46–6.99)	<0.001	2.28 (1.76–2.95)	<0.001	1.97 (1.52–2.57)	<0.001
Overlapping episode		1.98 (0.75–5.21)	0.17	1.82 (0.69–4.79)	0.23	1.95 (0.68–5.61)	0.22
Age		0.97 (0.96–0.98)	<0.001	0.98 (0.97–0.99)	<0.01	0.98 (0.97–1.00)	0.01
Sex	Female	Ref.					
	Male	1.51 (1.06–2.16)	0.02	1.35 (0.94–1.96)	0.11	1.36 (0.91–2.02)	0.14
Intellectual disability level	Mild	Ref.					
	Moderate	1.02 (0.66–1.59)	0.92	1.06 (0.67–1.66)	0.80	1.20 (0.73–1.98)	0.47
	Severe/profound	0.71 (0.41–1.22)	0.21	0.50 (0.26–0.94)	0.03	0.59 (0.29–1.17)	0.13
	Not specified/unknown	0.58 (0.38–0.90)	0.02	1.21 (0.77–1.91)	0.41	1.21 (0.74–2.00)	0.45
Ethnicity	White	Ref.					
	Black	0.87 (0.58–1.30)	0.49	0.88 (0.58–1.34)	0.55	0.78 (0.49–1.25)	0.30
	Asian	0.65 (0.31–1.34)	0.24	0.38 (0.17–0.84)	0.02	0.38 (0.16–0.90)	0.03
	Mixed	1.62 (0.69–3.83)	0.27	1.16 (0.50–2.71)	0.73	1.23 (0.49–3.10)	0.65
	Other	2.57 (0.98–6.77)	0.06	2.85 (1.12–7.24)	0.03	2.93 (1.06–8.14)	0.04
	Not stated	0.66 (0.28–1.52)	0.33	0.90 (0.39–2.07)	0.80	0.96 (0.39–2.34)	0.93
PDD diagnosis				1.64 (1.09–2.47)	0.02	1.84 (1.17–2.90)	<0.01
ADHD diagnosis				1.05 (0.53–2.09)	0.88	1.08 (0.51–2.30)	0.83
Ever diagnosed with a personality disorder				1.13 (0.41–3.13)	0.81	1.10 (0.36–3.33)	0.87
Ever diagnosed with SMI				1.37 (0.81–2.33)	0.24	1.37 (0.77–2.44)	0.29
Ever diagnosed with CMD				0.84 (0.52–1.34)	0.46	0.81 (0.48–1.37)	0.44
Physical health medication				0.93 (0.60–1.45)	0.76	0.85 (0.52–1.37)	0.50
Psychotropic medication				2.05 (1.31–3.19)	<0.01	2.26 (1.40–3.66)	<0.01
Total mentions of mood instability in episode				1.20 (1.02–1.40)	0.02	1.18 (1.00–1.40)	0.049
Total mentions of agitation in episode				1.43 (1.29–1.57)	<0.001	1.41 (1.28–1.56)	<0.001
Total mentions of irritability in episode				1.29 (1.09–1.53)	<0.01	1.29 (1.08–1.56)	<0.01
Total mental health professional contacts in episode						1.05 (1.02–1.09)	<0.001
Total mentions of social care package and/or home care package in episode						1.17 (1.00–1.37)	0.045

*Abbreviations*: ADHD, attention deficit hyperactivity disorder; CI, confidence interval; CMD, common mental disorder (depression and/or neurotic and stress-related disorders); OR, odds ratio; Other ethnicity groups, Arab and other minority ethnicity groups; PDD, pervasive developmental disorder diagnosis; Physical health medication, thyroid and antithyroid drugs, analgesics, and laxatives; Psychotropic medication, hypnotics/anxiolytics, antipsychotics, antidepressants, ADHD, and antiepileptic medication; SMI, severe mental illness (nonaffective psychotic disorders and/or bipolar/mania).

### Risk factors of adverse clinical outcome

Longer episodes were significantly associated with any of the adverse outcomes defined earlier in the unadjusted analysis (Model 1). When clinical variables were added in Model 2, we found statistically significant associations for having a personality disorder, CMD, greater number of mentions of agitation and episode length. In Model 3, adding service use, all of the previous variables remained as independent predictors of an adverse outcome as well as the greater number of contacts with professionals. In both Models 2 and 3, a diagnosis of PDD was associated with lower odds of a having any adverse outcome in those with aggression. That is people with PDD had fewer of the adverse outcomes as defined in this study. Details are shown in Table 3.

**Table 3.** Risk factors of adverse clinical outcomes in episodes with aggressive challenging behavior.

		Model 1		Model 2		Model 3	
		OR (95% CI)	*p*-value	OR (95% CI)	*p*-value	OR (95% CI)	*p*-value
Episode length in years		2.10 (1.65–2.66)	<0.001	1.51 (1.23–1.85)	<0.001	1.38 (1.12–1.71)	<0.01
Overlapping episode		2.85 (0.98–8.30)	0.06	1.96 (0.82–4.70)	0.13	2.07 (0.86–4.96)	0.10
Age		1.01 (1.00–1.02)	0.06	1.00 (0.99–1.01)	0.51	1.01 (1.00–1.02)	0.28
Sex	Female	Ref.					
	Male	0.71 (0.49–1.04)	0.08	0.89 (0.64–1.24)	0.50	0.8 (0.63–1.23)	0.47
Intellectual disability level	Mild	Ref.					
	Moderate	0.52 (0.32–0.84)	<0.01	0.74 (0.49–1.11)	0.14	0.80 (0.54–1.19)	0.27
	Severe/profound	0.55 (0.31–0.98)	0.043	0.78 (0.47–1.32)	0.36	0.88 (0.52–1.48)	0.64
	Not specified/unknown	0.86 (0.54–1.36)	0.52	1.21 (0.82–1.81)	0.34	1.25 (0.84–1.86)	0.27
Ethnicity	White	Ref.					
	Black	0.80 (0.52–1.23)	0.31	0.91 (0.63–1.33)	0.64	0.86 (0.59–1.27)	0.46
	Asian	1.33 (0.62–2.83)	0.46	1.01 (0.52–1.95)	0.98	1.07 (0.56–2.06)	0.84
	Mixed	0.62 (0.25–1.52)	0.30	0.53 (0.24–1.17)	0.11	0.52 (0.24–1.16)	0.11
	Other	1.86 (0.67–5.18)	0.23	1.36 (0.57–3.23)	0.49	1.35 (0.57–3.23)	0.50
	Not stated	0.29 (0.10–0.85)	0.03	0.39 (0.15–0.97)	0.043	0.40 (0.16–1.01)	0.05
PDD diagnosis				0.57 (0.38–0.86)	<0.01	0.59 (0.39–0.89)	0.01
ADHD diagnosis				1.14 (0.62–2.10)	0.67	1.20 (0.65–2.20)	0.56
Ever diagnosed with a personality disorder				2.79 (1.13–6.93)	0.03	2.82 (1.11–7.13)	0.03
Ever diagnosed with SMI				1.12 (0.72–1.74)	0.61	1.12 (0.72–1.74)	0.61
Ever diagnosed with CMD				1.88 (1.23–2.87)	<0.01	1.88 (1.22–2.89)	<0.01
Physical health medication				1.26 (0.87–1.81)	0.22	1.23 (0.86–1.77)	0.25
Psychotropic medication				1.25 (0.85–1.85)	0.26	1.28 (0.86–1.91)	0.22
Total mentions of mood instability in episode				1.06 (0.95–1.18)	0.32	1.03 (0.93–1.15)	0.56
Total mentions of agitation in episode				1.06 (1.03–1.10)	<0.001	1.04 (1.01–1.08)	0.01
Total mentions of irritability in episode				1.07 (0.98–1.17)	0.11	1.06 (0.98–1.16)	0.16
Total mental health professional contacts in episode						1.02 (1.01–1.04)	<0.01
Total mentions of social care package and/or home care package in episode						1.06 (0.96–1.16)	0.25

*Abbreviations*: ADHD, attention deficit hyperactivity disorder; CI, confidence interval; CMD, common mental disorder (depression and/or neurotic and stress-related disorders); OR, odds ratio; Other ethnicity groups, Arab and other minority ethnicity groups; PDD, pervasive developmental disorder diagnosis; Physical health medication, thyroid and antithyroid drugs, analgesics, and laxatives; Psychotropic medication, hypnotics/anxiolytics, antipsychotics, antidepressants, ADHD, and anti-epileptic medication; SMI, severe mental illness (nonaffective psychotic disorders and/or bipolar/mania).

## Discussion

### Main findings

We found that aggressive challenging behavior occurs in most episodes of clinical care requiring input from a community intellectual disability team. Exhibiting medium-high levels of aggressive challenging behavior was associated with increased length of care episode, being younger, having a PDD diagnosis, having mood instability, irritability, and agitation, increased contacts with mental health professionals, and increased mentions of a social care and/or home care package in the clinical notes. As may be expected, episodes with at least one mention of displaying aggressive challenging behavior also had more contact with mental health professionals and an increase in length of care episode.

Clinical risk factors associated with adverse outcomes included a diagnosis of CMD, personality disorder, and more mentions of agitation during the episode which indicates that they are part of a common presenting pathway of aggressive challenging behavior in people with intellectual disability. A diagnosis of PDD was associated with a decreased likelihood of adverse outcomes after adjusting for other risk factors. The quantity of the social care provided, though very important to families and providers, did not appear to be an independent risk for adverse outcome contrary to the finding of increased professional contacts. A recently published United Kingdom government action plan for maintaining community care for people with intellectual disability and autistic people who display challenging behavior [22] places a “good home,” “robust community support,” and “innovative local solutions” at the heart of reducing hospital care and mental health crises.

### Association with comorbidities

#### Mental ill-health

Mental illness in people with intellectual disability can be difficult to accurately diagnose due to atypical behavioral manifestations of disorders and hence a high level of diagnostic uncertainty can exist. A recent population-based study of 142 people with intellectual disability showed that almost one-third of participants in congregate settings had an undiagnosed mental disorder, with major depressive and anxiety disorders most common [23]. CMD were found to be an important predictor of adverse outcome in this study, suggesting a need for optimization of ascertainment and treatment of underlying mental ill-health to improve outcomes.

Mood instability, agitation, and irritability were associated with more mentions of aggressive challenging behavior. Such transdiagnostic symptoms may indicate the underlying mechanisms that result in aggressive challenging behavior and could be targets for specific intervention, rather than following purely diagnosis-driven treatment algorithms. The importance of these symptoms has been relatively under-reported in studies of adults with intellectual disability presenting with any challenging behavior, whereas they have been extensively explored in children and young people without intellectual disability [24, 25]. Symptoms of mood dysregulation and/or affective mental illness have been identified as predictors of physical aggression [10] and in one study as a predictor of readmission in those with intellectual disability and autism [9]. Of the episodes that had at least one mention of aggressive challenging behavior, a diagnosis of personality disorder was associated with adverse clinical outcomes. Personality disorder as a risk factor for aggressive challenging behavior in people with intellectual disability has not been well explored in the literature and is an area that warrants further investigation. It was recently shown to be a risk factor to readmission for people with intellectual disability and autism [9], supporting our findings that personality disorder and comorbid presentations of aggressive challenging behavior may lead to adverse outcomes.

#### PDD and ADHD

Neurodevelopmental disorders such as PDD and ADHD were more common as frequency of mentions of aggressive challenging behavior increased and almost 60% of those in the high aggression group had a co-occurring PDD. Autism has previously been identified as being associated with challenging behavior [11, 12], which we have confirmed in this study. However, we also found a decreased likelihood of adverse outcomes in those with a PDD diagnosis, which appears counterintuitive. This may represent the effects of a relatively recent widespread focus on behavioral aspects of care in the United Kingdom using interventions such as positive behavior support (PBS) to address environmental triggers, although a recent trial of PBS for challenging behavior did not demonstrate differential effectiveness in those with comorbid PDD [26].

ADHD is increasingly recognized in adults with intellectual disability and may underlie some presentations of challenging behavior [6, 27], however, can present differently in those with intellectual disability and standard diagnostic criteria may be less applicable in this population [28]. ADHD is a treatable condition [29]; screening and access to assessments with specialists are necessary to avoid diagnostic overshadowing and ensure appropriate management.

#### Physical health

Prescription of physical health medications as a proxy for physical illness was associated with higher frequency of aggressive challenging behavior. This suggests an association between physical illness and challenging behavior and highlights the need to proactively monitor and respond to early indicators of physical health problems; annual learning disability health checks are one such initiative [30]. There is also the possibility that some medications for physical health conditions could contribute to challenging behavior by causing adverse side effects which the person may find difficult to interpret or express verbally. Structured medication review at defined intervals and including collateral information from carers, have been suggested but evidence on patient outcomes has not been shown [31, 32].

#### Contacts with mental health professionals

Patients with more mentions of aggressive challenging behavior had a higher number of contacts with health professionals, including nurses, psychologists, and psychiatrists. Our results also demonstrate that more contacts with health professionals were significantly associated with increased odds of presenting with medium-high levels of aggressive challenging behavior as well as adverse clinical outcome after adjusting for other covariates. While this is to be expected, escalation in contacts could be potentially used by services to stratify risk within a caseload, similar to dynamic tools used to predict risk of admission [33]. Furthermore, it points to the high service costs and resource intensity that is required by such presentations, especially at times of mental health crisis.

#### Social care and home care packages

The quality and intensity of social support for people with intellectual disability who display aggressive challenging behavior is important in promoting well-being and improving mental and physical health. More mentions of a social and/or home care package were associated with medium-high levels of aggressive challenging behavior in episode, likely reflecting concern about the adequacy of care provision, or a need to review support. These findings suggest that discussions relating to social and/or home care package during an episode may serve as a useful indicator of patients who require more support or intervention. Other research has identified quality of care and the organizational environment within care settings are important aspects of the management of broadly defined challenging behavior in people with intellectual disability [34]. Research in this area is currently limited and further investigation of the constituent parts of social care support is needed to fully understand its impact in precipitating a recurrence or aid the recovery following an episode of aggressive challenging behavior.

### Strengths and limitations

This study utilized clinical data collected in specialist intellectual disability services, using a validated method for identifying aggressive challenging behavior in an automated way, which enabled the largest United Kingdom-based sample to be included. The use of routinely collected clinical data has allowed an insight into the significant personal and (indirect) service cost of aggressive challenging behavior and highlights the need for further investigation of correlates of aggressive challenging behavior and tailored interventions. The study sample was obtained from a major mental healthcare provider covering a diverse inner-city population and is, at least, generalizable to other urban areas serving similar populations. There is some overlap (e.g., presence of ADHD, presence of frustration, and mood lability) between our study and the other two comparable studies in the United Kingdom [6], but also several differences in that neither study reported likelihood of adverse outcomes nor service use as potential indicators of need. The present study includes a larger sample than either of the previous ones and therefore able to detect small but important effects.

Limitations include lack of causation. The type of aggressive challenging behavior and intensity were not distinguished in this study. Episodic severity, defined as the measure of intensity of a behavioral incident may be an important factor as it indicates the degree to or speed with which a behavioral incident can be safely resolved, but there is little evidence for its contribution to the management of aggressive challenging behavior [35]. We were also unable to disaggregate the detail of care packages in terms of hours provided and constituent parts. It is possible that not all people with intellectual disability who display aggressive challenging behavior or mental illness were referred to mental health services, and we did not include those who may have been seen in mainstream services, although we consider this number is likely to be very small. Finally, while data were collected from a diverse urban community, the findings of our findings originate from a United Kingdom setting and may not be replicated in other settings or countries with differing policies for treating people displaying aggressive challenging behavior.

## Conclusion

Our findings underline the importance of considering aggressive challenging behavior as a public health issue which needs further research and clinical investment, and more effective forms of individualized supportive intervention [36–38]. This should continue to be a priority for health and social care services owing to the impact of aggression on individuals with intellectual disability and their families and carers and the imposition of restrictive practices in response to challenging behavior [39]. Optimizing treatment of CMDs and personality disorder, and providing adequate and proactive support could reduce crisis presentations and the need for costly interventions including hospital admission.

## Data Availability

The data that support the findings of this study are available from the corresponding author, A.S., upon reasonable request.
